# Childhood hydrocephalus – is radiological morphology associated with etiology

**DOI:** 10.1186/2193-1801-2-11

**Published:** 2013-01-12

**Authors:** Jon Foss-Skiftesvik, Morten Andresen, Marianne Juhler

**Affiliations:** The Department of Neurosurgery Rigshospitalet University Hospital, Blegdamsvej 9, Copenhagen, 2100 Denmark

**Keywords:** Congenital hydrocephalus, Magnetic resonance imaging, Computed tomography, Aqueduct stenosis, Hydrocephalus classification

## Abstract

**Background:**

Clinicians use a non-standardized, intuitive approach when correlating radiological morphology and etiology of hydrocephalus.

**Objective:**

To investigate the possibility of categorizing hydrocephalus in different groups based on radiological morphology, to analyze if these proposed groups relate to the location and type of underlying pathology, and if this can be of use in clinical practice.

**Methods and material:**

A retrospective cohort study including 110 hydrocephalus patients below age seven seen at Rigshospitalet University Hospital, Denmark. Their neuro-imaging was analyzed and categorized based on radiological morphology. Patient charts were reviewed and possible association between the underlying cause of hydrocephalus and the proposed groups of radiological morphology was evaluated.

**Results:**

Radiological appearance varied distinctively between patients. A classification system was created based on the morphology of the lateral ventricles from axial sections at the level of maximal ventricular width. No statistically significant association was found between the suggested groups of morphology and the location and type of pathology.

**Conclusion:**

Distinguishable patterns of radiological morphology exist. The proposed classification system cannot in its current form indicate type and location of the underlying cause of hydrocephalus. A clear need exists for a standardized approach when evaluating etiology and treatment options based on radiological results.

## Background

Hydrocephalus is a complex neurological disorder characterized by an increased amount of cerebrospinal fluid (CSF) and enlargement of the cerebral ventricles and/or the subarachnoid space Rekate (2003). With a prevalence of 4.65 per 10 000 births Garne et al. (2009), congenital hydrocephalus is a common neurological diagnosis in children caused by flow obstruction(s), insufficient drainage or excessive CSF production. No international consensus exists concerning classification of hydrocephalus, and a number of different systems are currently in use Rekate (2011; Oi (2011), including cataloging based on age of onset, CSF dynamics and location of CSF accumulation, intracranial pressure (ICP) levels and the presence of symptoms. The most common causes of childhood hydrocephalus vary with age of onset, and include congenital malformation, tumor, cystic (mal-)formation, infection and hemorrhage Tsitouras & Sgouros (2011); Cinalli et al. (2011); Chatterjee & Chatterjee (2011); Garne et al. (2009).

Today, congenital hydrocephalus is diagnosed either by prenatal ultrasound (US) and genetic analysis or post partum by its characteristic clinical presentation combined with US, or more commonly computed tomography (CT) scanning or magnetic resonance imaging (MRI) Cinalli et al. (2011). CT and MRI technologies are therefore a central part of diagnosing, and also the evaluation of treatment options, follow-up and monitoring of patients Dincer & Ozek (2011).

When reviewing the initial neuro-imaging of hydrocephalus patients, it is our experience that many clinicians tend to take on a rather non-standardized, intuitive approach when correlating the radiological morphology with etiology. The point of transition from dilation to non-dilation of CSF spaces has previously been suggested as CT criterion for localizing an obstruction Naidich et al. (1982), but according to Cinalli and coworkers this may be misleading as up to 25% of non-obstructive hydrocephalus patients present with CT-scanning results showing little or no dilation of the fourth ventricle Cinalli et al. (2011).

The aim of this study is to investigate the possibility of categorizing hydrocephalus in different groups purely based on radiological morphology, to attain knowledge on whether these proposed groups relate to the different etiologies and the location of obstructive lesions, and if these possible associations can be of use in clinical practice.

## Results

### Groups of radiological morphology

**Figure 1:**
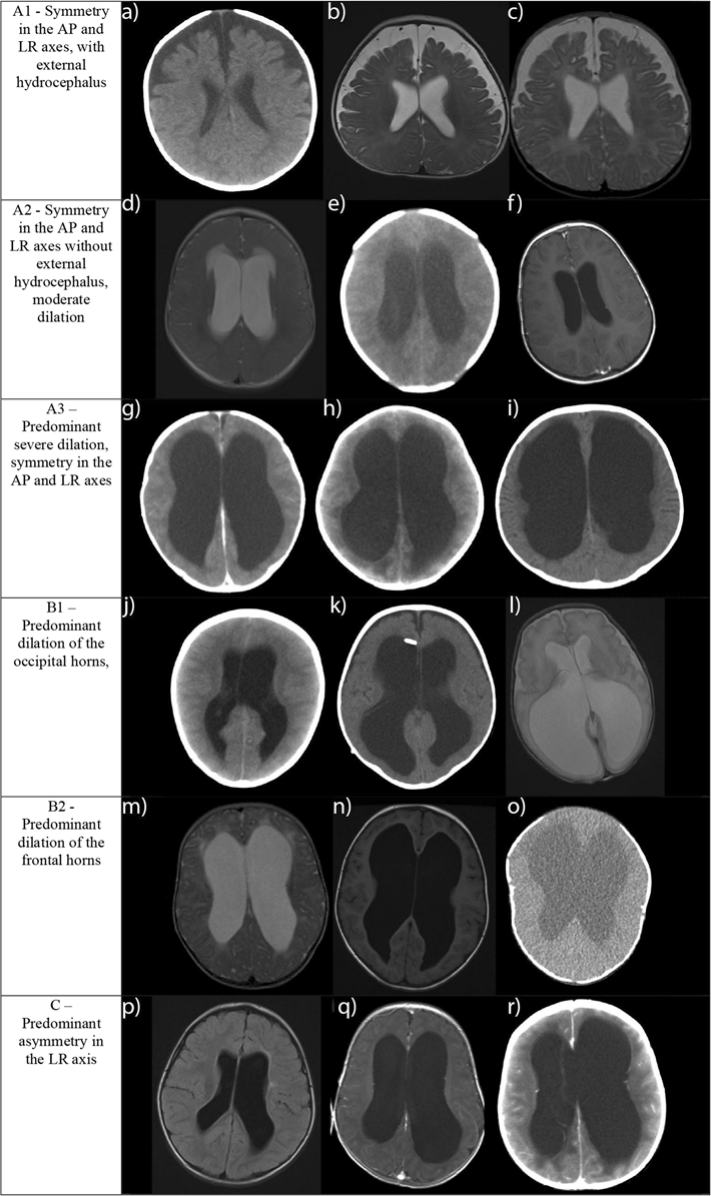
**Representative examples of the groups of radiological morphology.** AP: Anterio-posterior, LR: Left-to-right **a**) CT of a 3 month old boy with hydrocephalus of unknown cause, **b**) MRI of a 6 month old boy with hydrocephalus caused by Dandy-Walker malformation, **c**) MRI of a 5 month old boy with hydrocephalus of unknown cause, **d**) MRI of a 12 month old girl with hydrocephalus caused by brain stem tumor, **e**) CT of a 1 month old boy with hydrocephalus caused by meningitis, **f**) MRI of a 15 month old girl with hydrocephalus caused by brain stem tumor, **g**) CT of a 3 month old boy with hydrocephalus of unknown cause, **h**) CT of a 1 month old girl with hydrocephalus caused by hemorrhage, **i**) CT of a 16 month old boy with hydrocephalus caused by Dandy-Walker malformation, **j**) CT of an 11 month old boy with hydrocephalus of unknown cause, **k**) CT of a 4 month old boy with hydrocephalus caused by hemorrhage, **l)** MRI of a 1 month old girl with hydrocephalus caused by cystic malformation, **m**) MRI of a 6 month old girl with hydrocephalus caused by cystic malformation, **n**) MRI of a 38 month old girl with hydrocephalus of unknown cause, **o**) CT of a 2 month old boy with hydrocephalus caused by craniofacial malformation, **p**) MRI of a 19 month old girl with hydrocephalus of unknown cause, **q**) MRI of a 3 month old boy with hydrocephalus caused by post infectious cerebellitis, **r**) CT of an 11 month old boy with hydrocephalus caused by cystic malformation.

Based on the radiological appearance of the representative axial sections of the lateral ventricles, seven different morphological groups were constructed (Table 1 and Figure 1).

**Table 1:** **Description of proposed groups of radiological morphology, including their respective frequencies, percentages, mean EI and SD**

Groups of radiological morphology	Frequency (%)	Mean EI (SD)
A1 - Symmetry in the AP and LR axes, with external hydrocephalus	40 (36.4%)	0.337 (0.055)
A2 - Symmetry in the AP and LR axes without external hydrocephalus, moderate dilation	14 (12.7%)	0.340 (0.052)
A3 - Predominant severe dilation, symmetry in the AP and LR axes	12 (10.9%)	0.616 (0.060)
B1 – Predominant dilation of the occipital horns	20 (18.2%)	0.431 (0.078)
B2 - Predominant dilation of the frontal horns	3 (2.7%)	0.628 (0.052)
C – Predominant asymmetry in the LR axis	16 (14.5%)	0.498 (0.103)
X – Not categorized	5 (4.5%)	
Total	110 (100%)	

AP: Anterio-posterior, LR: Left-to-right, EI: Evan’s index, SD: Standard deviation.

As the different groups of radiological morphology, type of etiology and location of pathology comprised a limited number of patients, an assumption of normal distribution of Evan’s Index (EI) in the mentioned groups was concluded.

When examining the proposed groups of radiological morphology, group B is seen to contain both patients with symmetry and asymmetry in the left-to-right (LR) axis. Grouping these cases in two categories was abandoned as the dilation of the occipital horns was considered the predominant feature.

### Type and location of etiology

Overviews of the predefined groups of underlying causes, anatomical location of such and their respective frequencies are shown in Tables 2 and 3, respectively.

**Table 2:** **Overview of predefined types of etiology, their respective frequencies, percentages, mean EI and SD**

Type of etiology divided in subgroups	Frequency (%)	Mean EI (SD)
Tumor	13 (11.8%)	0.393 (0.120)
Cystic (mal-)formation(s) incl. Dandy Walker and Blake’s Pouch	12 (10.9%)	0.427 (0.164)
Hemorrhage(s)	20 (18.2%)	0.498 (0.131)
*Congenital malformation*	*27 (24,5%)*	*0.404 (0.111)*
Aqueduct stenosis	4 (3.6%)	0.506 (0.123)
Myelomeningocele with/without Chiari malformation	12 (10.9%)	0.375 (0.064)
Chiari malformations without myelomeningocele	1 (0.9 %)	0.561
Craniofacial malformation	10 (9.1%)	0.379 (0.125)
Infection and post-infectious inflammation	6 (5.5%)	0.469 (0.076)
Trauma	3 (2.7%)	0.326 (0.051)
Venous pathology	4 (3.6%)	0.317 (0.033)
Unknown	25 (22.7%)	0.385 (0.092)
Total	110	

EI – Evan’s Index, SD – Standard deviation.

**Table 3:** **Overview of predefined groups of anatomical location of etiology, their respective frequencies, percentages, mean EI and SD**

Location of etiology divided in subgroups	Frequency (%)	Mean EI (SD)
*Cerebrum*	*62 (56,2)*	*0.442 (0.132)*
Supratentorial	30 (27.3%)	0.489 (0.137)
Sylvian aqueduct	5 (4.5%)	0.506 (0.107)
Brain stem and corpus pineale	4 (3.6%)	0.340 (0.023)
Posterior fossa	14 (12.7%)	0.394 (0.134)
Meningeal	4 (3.6%)	0.450 (0.090)
Unspecified	5 (4.5%)	0.359 (0.103)
Spinal cord	12 (10.9%)	0.375 (0.064)
Craniofacial	11 (10%)	0.372 (0.121)
Unknown	25 (22.7%)	0.385 (0.092)
Total	110 (100%)	

EI – Evan’s Index, SD – Standard deviation.

### Association between etiology and radiological morphology

#### Type of etiology

No clear link was found when analyzing the relationship between type of etiology and groups of radiological morphology (see Table 4). Grouping all congenital malformations together did not change this observation. When analyzing cross tables showing one type of etiology vs. all others, the only two types of etiologies presenting with significant difference in groups of radiological morphology were craniofacial malformation and hemorrhage. 90% of the patients with craniofacial malformation were found to have symmetry in both the anterior-posterior (AP) and LR axes (therefore listed in group A1 or A2).

**Table 4:** **Overview of how types of etiology (rows) are distributed between the groups of radiological morphology (columns)**

		Groups of radiological morphology							
		A1 – Symmetry in the AP and LR axes, with external hydrocephalus	A2 - Symmetry in the AP and LR axes without external hydrocephalus, moderate dilation	A3 – Predominant severe dilation, symmetry in the AP and LR axes	B1 – Predominant dilation of the occipital horns	B2 – Predominant dilation of the frontal horns	C – Predominant asymmetry in the LR axis	X – Not categorized	Total
**Types of Etiology**	Tumor	**3** (23.1%)^a^	**5** (38.5%)	**2(**15.4%)	**1** (7.7%)	**0** (0%)	**1** (7.7%)	**1** (7.7%)	**13**(100%)
		(7.5%)^b^	(35.7%)	(16.7)	(5%)	(0%)	(6.2%)	(20%)	(11%)
	Cystic (mal-) formation(s)	**3** (25%)	**0** (0%)	**2(**16.7%)	**3** (25%)	**1** (8.3%)	**3** (25%)	**0** (0%)	**12**(100%)
		(7.5%)	(0%)	(16.7)	(15%)	(33.3%)	(18.8%)	(0%)	(11%)
	Hemorrhage	**3** (15%)	**0** (0%)	**4** (20%)	**6** (30%)	**0** (0%)	**3** (15%)	**4** (20%)	**20(**100%)
		(7.5%)	(0%)	(33.3%)	(30%)	(0%)	(18.8%)	(80%)	(18.3%)
	Congenital malformation								
	*Aqueduct stenosis*	**0** (0%)	**0** (0%)	**1** (25%)	**3** (75%)	**0** (0%)	**0** (0%)	**0** (0%)	4(100%)
		(0%)	(0%)	(8.3%)	(15%)	(0%)	(0%)	(0%)	(3.7%)
	*MMC with/without Chiari*	**4** (33.3%)	**3** (25%)	**0** (0%)	**4** (33.3%)	**0** (0%)	**1** (8.3%)	**0** (0%)	**12**(100%)
		(10%)	(21.4%)	(0%)	(20%)	(0%)	(6.2%)	(0%)	(11%)
	*Chiari w/o MMC*	**0** (0%)	**0** (0%)	**0** (0%)	**1** (100%)	**0** (0%)	**0** (0%)	**0** (0%)	1**(**100%)
		(0%)	(0%)	(0%)	(6.2%)	(0%)	(0%)	(0%)	(0.9%)
	*Craniofacial*	**5** (50%)	**4** (40%)	**0** (0%)	**0** (0%)	**1** (10%)	**0** (0%)	**0** (0%)	**10**(100%)
		(12.5%)	(28.6%)	(0%)	(0%)	(33.3%)	(0%)	(0%)	(9.2%)
	Infection/post-infectious inflammation	**1** (16.7%)	**1** (16.7%)	**0** (0%)	**0** (0%)	**0** (0%)	**4** (66.7%)	**0** (0%)	6**(**100%)
		(2.5%)	(7.1%)	(0%)	(0%)	(0%)	(25%)	(0%)	(5.5%)
	Trauma	**2** (66.7%)	**0** (0%)	**0** (0%)	**1** (33.3%)	**0** (0%)	**0** (0%)	**0** (0%)	**3**(100%)
		(5%)	(0%)	(0%)	(5%)	(0%)	(0%)	(0%)	(2.8%)
	Venous pathology	**3** (75%)	**0** (0%)	**0** (0%)	**0** (0%)	**0** (0%)	**1** (25%)	**0** (0%)	**4**(100%)
		(7.5%)	(0%)	(0%)	(0%)	(0%)	(6.2%)	(0%)	(3.7%)
	Unknown	**16** (64%)	**1** (4%)	**3** (12%)	**2** (8%)	**1** (4%)	**2** (8%)	**0** (0%)	**25**(100%)
		(40%)	(7.1%)	(25%)	(10%)	(33.3%)	(12.5%)	(0%)	(22.9%)
	Total	**40** (36.4%)	**14** (12.7%)	**12**(10.9%)	**20** (18.2%)	**3** (2.7%)	**16** (14.5%)	**5** (4.5%)	**110**(100%)
		(100%)	(100%)	(100%)	(100%)	(100%)	(100%)	(100%)	(100%)

^a^ – % within type of etiology, ^b^ - % within groups of radiological morphology. MMC – myelomeningocele, AP – anterioposterior, LR – left-to-right.

#### Location of etiology

A similar lack of pattern is seen when studying the possible connection between location of etiology and groups of radiological morphology (see Table 5). 80% of all patients with pathology located in the Sylvian aqueduct showed predominant dilation of the occipital horns (group B1), but only 20% of the morphological group as a whole had this location of pathology.

**Table 5:** **Overview of how location of etiology (rows) is distributed between the groups of radiological morphology (columns)**

		Groups of radiological morphology							
		A1 – Symmetry in the AP and LR axes, with external hydrocephalus	A2 - Symmetry in the AP and LR axes without external hydrocephalus, moderate dilation	A3 – Predominant severe dilation, symmetry in the AP and LR axes	B1 – Predominant dilation of the occipital horns	B2 – Predominant dilation of the frontal horns	C – Predominant asymmetry in the LR axis	X – Not categorized	Total
**Location of Etiology**	Cerebrum								
	*Supratentorial*	**7** (23.3%)^a^	**0** (0%)	**6**(20%)	**5** (16.7%)	**1** (3.3%)	**6** (20%)	**5** (16.7%)	**30**(100%)
		(17.5%)^b^	(0%)	(50%)	(25%)	(33.3%)	(37.5%)	(100%)	27.3%)
	*Sylvian aqueduct*	**0** (0%)	**0** (0%)	**1** (20%)	**4** (80%)	**0** (0%)	**0** (0%)	**0** (0%)	**5**(100%)
		(0%)	(0%)	(8.3%)	(20%)	(0%)	(0%)	(0%)	(4.5%)
	*Brainstem & corpus pineale*	**0** (0%)	**3** (75%)	**0** (0%)	**0** (0%)	**0** (0%)	**1**(25%)	**0** (0%)	**4**(100%)
		(0%)	(21.4%)	(0%)	(0%)	(0%)	(6.2%)	(0%)	(3.6%)
	*Posterior fossa*	**4** (28.6%)	**2** (14.3%)	**2**(14.3%)	**4** (28.6%)	**0** (0%)	**2** (14.3%)	**0** (0%)	**14**(100%)
		(10%)	14.3%)	(16.7%)	(20%)	(0%)	(12.5%)	(0%)	(12.7%)
	*Meningeal*	**1** (25%)	**1** (25%)	**0** (0%)	**0** (0%)	**0** (0%)	**2** (50%)	**0** (0%)	**4**(100%)
		(2.5%)	(7.1%)	(0%)	(0%)	(0%)	(12.5%)	(0%)	(3.6%)
	*Unspecified*	**2** (40%)	**0** (0%)	**0** (0%)	**1** (20%)	**0** (0%)	**2** (40%)	**0** (0%)	**5**(100%)
		(5%)	(0%)	(0%)	(5%)	(0%)	(12.5%)	(0%)	(4.5%)
	Spinal cord	**4** (33.3%)	**3** (25%)	**0** (0%)	**4** (33.3%)	**0** (0%)	**1** (8.3%)	**0** (0%)	**12**(100%)
		(10%)	(21.4%)	(0%)	(20%)	(0%)	(6.2%)	(0%)	(10.9%)
	Craniofacial	**6** (54.5%)	**4** (36.4%)	**0** (0%)	**0** (0%)	**1** (9.1%)	**0** (0%)	**0** (0%)	**11**(100%)
		(15%)	(28.6%)	(0%)	(0%)	(33.3%)	(0%)	(0%)	(10%)
	Unknown	**16** (64%)	**1** (4%)	**3** (12%)	**2** (8%)	**1** (4%)	**2** (8%)	**0** (0%)	**25**(100%)
		(40%)	(7.1%)	(25%)	(10%)	(33.3%)	(12.5%)	(0%)	(22.7%)
	Total	**40** (36.4%)	**14** (12.7%)	**12**(10.9%)	**20** (18.2%)	**3** (2.7%)	**16** (14.5%)	**4** (4.5%)	**110**(100%)
		(100%)	(100%)	(100%)	(100%)	(100%)	(100%)	(100%)	(100%)

^a^ – % within location of etiology, ^b^ - % within groups of radiological morphology. AP – anterior-posterior, LR – left-to-right.

#### Asymmetrical appearance

Distinct asymmetry of the lateral ventricles in the LR axis on imaging was observed in 25 patients. This also included patients from groups of radiological morphology where asymmetry in the LR axis was not considered the predominant feature. Reflecting on the before-mentioned theory of point of obstruction, an independent analysis of the association between lateral ventricle asymmetry and location of pathology was conducted (Table 6).

**Table 6:** **Cross table showing association between asymmetrical appearance on neuro-imaging and location of underlying pathology**

		Morphological appearance		
		Symmetrical	Asymmetrical	Total
**Location of Etiology**	Cerebrum	**43** (69.4%)^a^	**19** (30.6%)	**62** (100%)
		(50%)^b^	76%)	(56.4%)
	Non-Cerebrum	**21** (87.5%)	**3** (12.5%)	**24** (100%)
		(24.7%)	(12%)	(21.8%)
	Unknown	**21** (87.5%)	**3** (12.5%)	**24** (100%)
		(24.7%)	(12%)	(21.8%)
	Total	**85** (77.3%)	**25** (22.7%)	**110**(100%)
		(100%)	(100%)	(100%)

^a^ - % within location of etiology.

^b^ - % within morphological appearance.

The ratio between cerebral and non-cerebral pathology (including unknown) in patients presenting with LR asymmetry on neuro-imaging was 3.2:1. This trend was not statistically significant (p = 0.079). No such trend was observed when comparing supratentorial pathology to pathology of non-supratentorial or unknown location.

**Figure 2:**
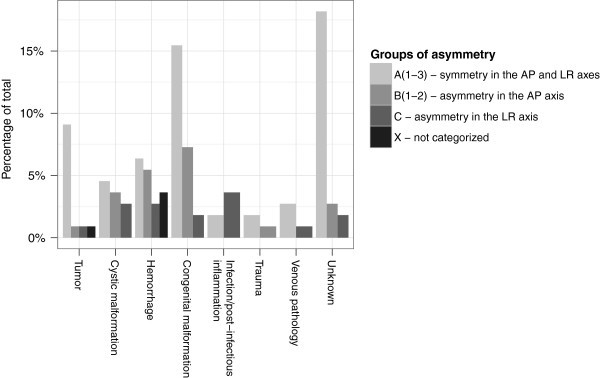
**Bar chart illustrating the distribution of patients with symmetry/asymmetry on neuro-imaging between the types of etiology.** AP – anterior-posterior, LR – left-to-right.

When analyzing how symmetry/asymmetry on neuro-imaging vary with different types of etiology, symmetry in both axes is seen to dominate except in patients with hydrocephalus caused by infection or post-infectious inflammation (see Figure 2). 66.7% of these patients presented with asymmetry in the LR axis, but only 25% of the morphological group as a whole suffered from infection-caused hydrocephalus.

#### Age stratification

The patients were divided in three groups based on age at time of initial scanning, respectively 0–1 months, 1.1-12 months and 12.1- ∞ months. No clear link was observed when correlating the age-stratified groups with type of etiology. 51.6% of patients between 1.1-12 months were found in group A1, representing 80% of this group of radiological morphology. No other relevant age related observations were made.

### Evan’s index

#### Groups of Radiological Morphology

**Figure 3:**
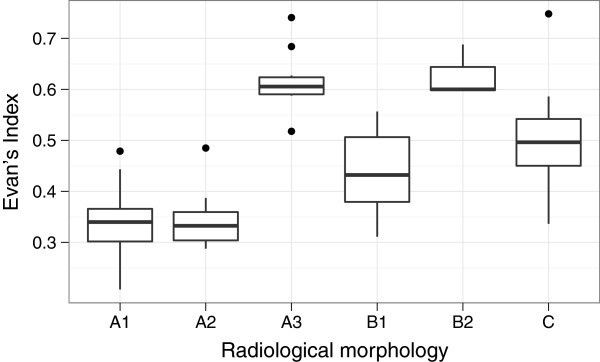
**Box plot illustrating the distribution of Evan’s Index between groups of radiological morphology.** A1 – Symmetry in the AP and LR axes, with external hydrocephalus A2 – Symmetry in the AP and LR axes without external hydrocephalus, moderate dilation A3 – Predominant severe dilation, symmetry in the AP and LR axes B1 – Predominant dilation of the occipital horns B2 – Predominant dilation of the frontal horns C – Predominant asymmetry in the LR axis AP – anterior-posterior, LR – left-to-right.

As expected, the EI showed statistically significant variation between the proposed groups of radiological morphology increasing with the dilation of the lateral ventricles (see Figure 3).

#### Types of etiology

More interestingly, the EI was found to vary between the different types of underlying causes (see Table 2). Though no statistically significant differences in EI were detected between the groups, there seems to exist a trend. While patients with hydrocephalus caused by tumor had a mean EI of 0.392, the group suffering from congenital stenosis of the Sylvian aqueduct presented with a mean EI of 0.506.

#### Location of etiology

EI was also found to differ depending on location of pathology (see Table 3). Patients with hydrocephalus caused by pathology located to the cerebrum presented with a mean EI of 0.437, while the mean EI of cases with pathology located to spinal cord, craniofacial or of unknown location varied from 0.375-0.379. The highest values of mean EI were found in patients with pathology located to the Sylvian aqueduct or supratentorially. The only statistically significant inter-group difference was shown between patients with supratentorial pathology and those with pathology of unknown location (p=0.045).

## Discussion

It is evident that distinguishable patterns of morphology in neuro-imaging exist between patients with hydrocephalus. What causes the varying appearance remains uncertain. The proposed arrangement of radiological morphology made by the authors is only one of many possible classification systems for CT and MRI appearance of this patient group.

This study found no association between groups of radiological morphology and underlying cause or location of the pathological lesion. Though no such association was observed, it is likely that the clearly different appearances of hydrocephalus on axial sections of the lateral ventricles have underlying patophysiological causes. These might include non-visualized obstructions, hemorrhages absorbed at time of imaging, genetic migration defects etc.

The observed high EI among patients with stenosis of the Sylvian aqueduct indicates pre-obstruction dilation, and therefore supports the theory that the point of transition from dilation to non-dilation can forecast the location of an obstruction. As the aqueduct itself and the distal parts of the ventricle system were not visualized in the study, this remains only an indication.

This also limited extrapolation of the theory to the patients with meningeal pathology, as EI is more sensitive to dilation of the anterior horns and as the fourth ventricle was not visualized. Whether patients with pathology located to the leptomeninges have a greater tendency to compensate by dilating their fourth ventricle or their cranio-cortical space is unknown. Only one out of four patients with meningeal pathology showed enlarged cranio-cortical width (CCW).

The fact that asymmetry of the lateral ventricles in the LR axis did not seem to be linked to supratentorial pathology indicates a lack of associated to an obstruction of the foramen Monro. This suggests that asymmetry can result from other causes than a closed foramen Monro.

Half of the patients between 1.1-12 months showed symmetry in both axes and external hydrocephalus. Although no link was seen between the different age groups and type of etiology, this might indicate the need to include age in a future radiological prediction tool. The size of this study’s patient population was a limiting factor in investigating this further.

The system for classification of radiological morphology in hydrocephalus patients proposed by the authors cannot in its current form be used to forecast underlying causes or locations of such pathology in clinical practice. This does not exclude the use of radiological morphology patterns as an aid in clinical practice in the future. The classification system presented in this study was based on axial sections at the level of largest lateral ventricle width. Future systems for classification of radiological morphology in hydrocephalus patients should probably comprise a combination of axial, coronal and saggital sections to create a 3D model of the ventricular system. Being able to observe the ventricular system as a whole, its possible obstructions and/or malformations, will aid in the understanding of association between etiology and radiological morphology.

## Conclusion

Hydrocephalus still remains a highly challenging condition in terms of both diagnosis and management. A clear need exists for a standardized approach when evaluating underlying causes and treatment options depending on radiological results. As many clinicians tend to base their choice of treatment on neuro-imaging, it is evident that the establishment of a classification system based on the association between underlying cause and radiological appearance will help in optimizing the treatment of hydrocephalus.

## Methods and material

### Study design

We conducted a retrospective cohort study including all patients diagnosed with hydrocephalus who received patient care at Rigshospitalet University Hospital, Copenhagen, Denmark, during the period between 01.01.2008 and 12.31.2011. A subset of patients below the age of seven was selected, thus including only infantile or early juvenile hydrocephalus patients. The database search included patients diagnosed at Rigshospitalet and patients referred with a tentative hydrocephalus diagnosis. Both in- and out-patient services were included, resulting in a total of 137 patients.

### Course of study

The primary imaging results of all patients were reviewed, not differentiating between CT and MRI techniques. If an MRI had been performed within 3 weeks of an initial CT scan, the MRI was included in the study instead, provided no surgical treatment had been initiated between the two procedures.

The radiological material was then analyzed with AgfaWeb1000 v5.1.2., and representative axial sections of the lateral ventricles were obtained at the level of maximal lateral ventricle width. This approach was chosen as a substantial number of hydrocephalus patients are often initially seen at facilities where only axial plane CT imaging is available. CT reconstructions of the sagittal and coronal planes are available at various radiology departments, but the often higher quality axial sections serve as the primary imaging material in clinical practice. The imaging results were categorized in groups purely based on their visual morphologic appearance, taking into consideration factors such as the size, shape, and symmetry of the AP and LR axes of the lateral ventricles. The EI was calculated for all patients. Although debated Moore et al. (2012); Ambarki et al. (2010); Pakkenberg et al. (1989); Penn et al. (1978), EI is still one of the most used quantitative measures among clinicians and therefore supported our choice of axial sections. It is important to remember that it can be a biased estimator for ventricular volume (VV) as its ordinary interpretation assumes that all ventricular systems share the same shape. Assessment of VV was not possible based on limitations in a majority of the imaging material.

The hydrocephalus etiology for each patient was investigated by reviewing patient charts. Underlying causes were categorized according to predefined groups of pathology and anatomic locations.

### Exclusion procedure

See Figure 4.

**Figure 4:**
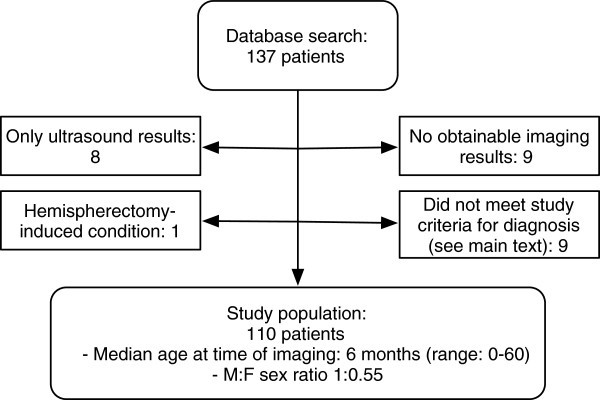
**Flowchart illustrating the exclusion procedure.**

15.5% of the patients had ventriculo-peritoneal (VP) shunts at the time of their first obtainable imaging result, with MRI available for 59%, and CT for 41%. VP treatment was not considered an exclusion criterion.

### Diagnostic criteria

The hydrocephalus diagnosis was confirmed using radiological parameters. Clinical features, such as an increase in head circumference, were not taken into account, as there is no current international agreement on including clinical symptoms in the diagnostic criteria Rekate (2009). As ventriculomegaly is defined by EI ≥ 0.3 Evans (1942), patients with EI <0.3 were excluded unless co-existence of external hydrocephalus was identified, disqualifying the above mentioned 9 patients from the study (see Figure 3).

External hydrocephalus is defined as a rapid increase in head circumference in an infant combined with an enlarged frontal subarachnoid space with normal or slightly enlarged ventricles as seen on CT, MRI or cranial US Zahl et al. (2011). No international consensus exists concerning limits of enlargement of the subarachnoid space, and the upper limits for CCW in infants (below one year of age) range from 3.3 to 10 mm Prassopoulos et al. (1995); Fessell et al. (2000); Lam et al. (2001); Libicher & Troger (1992); Frankel et al. (1998).

In this study, enlargement of the subarachnoid space was defined by a CCW of more than 4 mm in the fronto-temporal area, as shown with CT technology in a similar age group by Prassopoulos and coworkers.

### Statistical analysis

Statistical analysis was performed using SPSS software v20.0 (IBM Advanced Statistics SPSS version 20.0). Quantitative data were expressed as mean ± standard deviation (SD), qualitative data as frequencies and percentages. Comparison of categorical data was done by visual evaluation, chi-square and post-hoc exploratory analysis using Fisher’s exact test. A linear model with post-hoc Tukey Honest Significant Difference test was used when analyzing numerical values. P-values < 0.05 were considered statistically significant.

## Abbreviations

CSF: Cerebrospinal fluid
ICP: Intracranial pressure
US: Ultrasound
CT: Computed tomography
MRI: Magnetic resonance imaging
LR: Left-to-right
AP: Anterior-posterior
CCW: Cranio-cortical width
EI: Evan’s index
VP: Ventriculo-peritoneal (shunt)
SD: Standard deviation; Explanations are provided in the text when first used.
